# Ten-Year Outcomes of Intravitreal Bevacizumab for Myopic Choroidal Neovascularization: Analysis of Prognostic Factors

**DOI:** 10.3390/ph14101042

**Published:** 2021-10-13

**Authors:** Fabiana Mallone, Rosalia Giustolisi, Federica Franzone, Marco Marenco, Rocco Plateroti, Marcella Nebbioso, Alessandro Lambiase, Magda Gharbiya

**Affiliations:** Department of Organ of Sense, Sapienza University of Rome, 00185 Rome, Italy; fabiana.mallone@uniroma1.it (F.M.); rosalia.giustolisi@uniroma1.it (R.G.); federica.franzone@uniroma1.it (F.F.); marco.marenco@uniroma1.it (M.M.); rocco.plateroti@uniroma1.it (R.P.); marcella.nebbioso@uniroma1.it (M.N.); magda.gharbiya@uniroma1.it (M.G.)

**Keywords:** ocular pharmacology, bevacizumab (IVB), pathologic myopia (PM), myopic choroidal neovascularization (mCNV), ophthalmology, drug delivery systems, long-term results

## Abstract

The current standard treatment of myopic choroidal neovascularisation (mCNV) is intravitreal injection of VEGF antagonists. This study was proposed to assess efficacy and safety of intravitreal bevacizumab (IVB) for the treatment of mCNV across a 10-year follow-up. Thirty eyes of thirty patients with treatment-naïve mCNV who underwent IVB and were followed up with for a minimum of ten years were recruited for the present retrospective cohort study. All participants were treated with three monthly IVB at baseline and then evaluated and treated under pro re nata (PRN) schedule. Outcome measures were to determine BCVA changes over years and identify the predictive factors of both final visual outcome and need for retreatment. Analysis of the main involved prognostic factors with correlations among variables is reported. Visual acuity remained stable at 10-year follow-up (*p* = 0.001) with the greatest improvement at 2 years (*p* < 0.0001) in all CNV locations. Baseline BCVA correlated positively with final BCVA (β = 0.88, *p* < 0.0001, R^2^: 0.75). No predictive factors for the need of additional injections were identified. Retinal and choroidal thickness significantly reduced over time but without correlation with the number of injections. CNV max height and area significantly decreased at 10 years (*p* < 0.0001 and *p* = 0.003, respectively), with complete regression of mCNV lesion in 40% of subjects. Intravitreal bevacizumab resulted as long-term effective and safe therapy for mCNV with sustained results at 10 years.

## 1. Introduction

Myopic choroidal neovascularization (mCNV) is the most frequent sight-threatening complication of pathological myopia (PM), occurring with an estimated incidence of nearly 10.2% and accounting for as much as 62% of CNV in patients under the age of 50. CNV secondary to myopia represents the leading cause of progressive central visual loss in working-age subjects, with over 90% of affected eyes developing legal blindness [1,2]. In addition, the affected individuals present a greater risk of developing mCNV in the fellow eye (34.8%) within approximately 8 years, if compared to patients without pre-existing CNV (6.1%) [1].

At present, intravitreal injections of antivascular endothelial growth factor (anti-VEGF) are recommended as first-line therapy for mCNV [3,4]. Previous therapies such as laser photocoagulation for extra/juxtafoveal mCNV and photodynamic therapy with verteporfin for subfoveal mCNV turned out to be unsatisfactory in the long term [5]. Intravitreal bevacizumab (IVB) (Avastin; Genentech, South San Francisco, CA, USA) produced convincing short-term outcomes in mCNV, with no reported serious ocular or systemic side effects [6,7,8,9,10]. Several studies in the literature evaluated the long-term efficacy of IVB; however, conflicting results on BCVA changes over time and limited instrumental data were presented. Additionally, differences in the baseline characteristics of patients (previous treatments, older age of the recruited patients), therapeutic protocol (either one single or three monthly intravitreal injections with pro re nata (PRN) as the follow-up protocol) and follow-up time (from 2- to 6-year evaluations) questioned the reliability of these papers and made further investigation necessary [11,12,13,14,15,16,17,18,19,20,21,22,23,24,25,26,27].

The present retrospective cohort study is intended to evaluate the long-term efficacy and safety of IVB for the treatment of mCNV over 10 years follow-up time. Administration of three monthly IVB at baseline and then rigorous monthly monitoring under PRN protocol was scheduled. Analysis of the main involved prognostic factors and correlations among variables is discussed.

## 2. Results

### 2.1. Demographic and Clinical Data

Thirty eyes of thirty consecutive patients with mCNV treated with IVB were studied. All CNVs were classified as classic/type 2 on FA and SD-OCT according to Freund et al. [28]. The mean age was 63.7 ± 7.6 with a range of 49 to 74 years, and the mean axial length was 30.7 ± 1.2 mm with a range of 29.1 to 32.2 mm. The majority of mCNV were juxtafoveal (46.7%) with subfoveal and extrafoveal location respectively observed in 20% and 33.3% of all cases. Need for retreatment was estimated at 46.7% (14 eyes), with 4 eyes receiving additional injections for CNV persistence and 10 eyes receiving additional injections for CNV recurrence. Sixteen eyes (53.3%) only received the loading dose of three monthly IVB injections. We found 26.7% of cases (eight patients) developed mCNV in the fellow eye, with presentation at approximately 7 years in three patients and 1 year for the remaining five patients. The mean total number of anti-VEGF injections was 6.8 ± 6.31 (range 3–24) over the 10-year study period, and the majority administered in the first year of treatment (mean 5.1 ± 3.36). Demographic and clinical data are listed in Table 1. Chorioretinal atrophy (CRA) developed and progressed around the mCNV in 24 eyes (80%) at 3 years and in all study eyes at 5 and 10 years after the onset of CNV, based on instrumental evaluations (colour fundus photography, FA and OCT images). Data on number of IVB injections and incidence of CRA during the 10-year follow-up period are summarized in Table 2.

### 2.2. BCVA Outcomes

The 10-year follow-up was completed for all patients, with the greatest improvement of BCVA at 2 years (*p* < 0.0001) and sustained results at the 10-year follow-up (*p* = 0.001). Mean (±SD) baseline BCVA was 0.47 log MAR (±0.42). After treatment, it was 0.28 (±0.33) log MAR at 2 years and 0.21 (±0.29) log MAR at 10 years. Visual results over time are detailed in Table 3. Multivariate regression analysis showed that baseline BCVA was the only pretreatment variable significantly correlated with BCVA outcome (β = 0.88, *p* < 0.0001). The adjusted R^2^ of the final model was 0.75 at 10 years (Table 4). Interestingly, we did not find any correlation between final visual outcome and CNV location.

### 2.3. OCT and FA Outcomes

**Figure 1:**
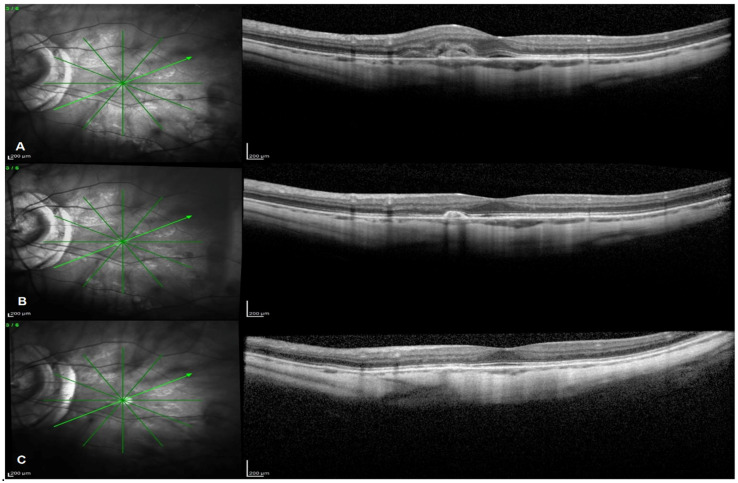
Spectral domain optical coherence tomography (SD-OCT) consecutive images (**A**–**C**) referring to baseline, 5-year and 10-year follow-up times of a 49-year-old female patient suffering from mCNV and treated with IVB (radial 20°, six lines scan protocol centred on the fovea). (**A**) Baseline scan showing an active juxtafoveal type 2 mCNV with macular thickening and minimal subretinal fluid. (**B**) Year 5 scan showing a residual highly reflective subretinal scar lesion with no associated fluid. (**C**) Year 10 scan showing complete regression of the mCNV lesion.

Mean CNV max height decreased from 165.53 (±108.27) µm at baseline to 72.4 (±67.44) µm at 5 years (*p* < 0.0001) and to 59.53 (±61.29) µm at 10 years (*p* < 0.0001). Mean CNV area decreased from 0.12 (±0.11) mm^2^ at baseline to 0.05 (±0.06) mm^2^ at 5 years (*p* = 0.017) and to 0.04 (±0.06) mm^2^ at 10 years (*p* = 0.003) with complete regression of mCNV lesion in 40% of subjects (Figure 1). Mean CSF choroidal thickness significantly decreased from baseline to fifth year (*p* = 0.029) and from baseline to year 10 (*p* = 0.036). Similar results were obtained for mean choroidal thickness at 3 mm and 6 mm distance from the fovea at the 5- and 10-year follow-ups. A significant decrease in mean CSF retinal thickness was observed between baseline and year 5 (*p* = 0.01), between year 5 and year 10 (*p* = 0.038) and between baseline and year 10 (*p* = 0.007). Additionally, mean RT at 3 mm significantly decreased from baseline to year 5 (*p* = 0.014), from year 5 to year 10 (*p* = 0.035) and from baseline to year 10 (*p* = 0.003), whereas mean RT at 6 mm significantly decreased from year 5 to year 10 (*p* = 0.027) and from baseline to year 10 (*p* = 0.011). Anatomical outcomes are presented in Table 5. A comparative analysis between patients and a group of 30 subjects affected by uncomplicated PM and followed-up with for at least 10 years did not reveal any significant difference in both choroidal and retinal thickness change (Table 6).

No correlation was demonstrated between retinal and choroidal thickness over time and mean total number of injections. Statistically significant correlation was observed between baseline CSF choroidal thickness and baseline CNV size (*p* = 0.041; r = 0.533) and height (*p* = 0.034; r = 0.549), but this result was not maintained at the end of follow-up.

No predictive factors for the need for retreatment were identified. Absence of leakage on FA and of intra/subretinal fluid on OCT was demonstrated in 26 of the 30 eyes (86.7%) at 5 years and in all 30 eyes (100%) at the end of follow-up. The intraobserver and interobserver agreement in instrumental assessment was excellent (Kappa = 0.874).

No ocular injection-related adverse events were observed. None of the patients enrolled experienced any systemic complications during the whole follow-up period.

## 3. Discussion

Myopic CNV is the most common and severe complication of PM, and anti-VEGF therapy is actually the gold standard treatment [3]. Several studies have reported the short-term efficacy and safety of intravitreally administered bevacizumab [6,7,8,9,10]. Favourable results emerged regardless of the different employed dosages (from 1 to 2.5 mg), and the adopted protocols (nonloading dose treatment vs. three monthly injections, followed by an as-needed schedule for the subsequent period). Unlike the positive outcomes of studies with relatively short follow-up time, longer follow-up results are conflicting in mCNV.

The present study demonstrated sustained functional and morphological results of IVB therapy for mCNV over 10 years of follow-up time. All participants were treated with the same standardized schedule: three monthly IVB 1.25 mg at baseline, and then evaluated and treated under PRN, with strict monthly monitoring of disease activity under clinical and instrumental evaluation. The impact of previous PDT treatment was not evaluated since we only recruited treatment naïve mCNV patients.

The primary outcome measure of the present study was the mean change in BCVA from baseline to year 10. Particularly, IVB therapy significantly improved BCVA at 2 years (*p* < 0.0001) in mCNV, with visual acuity remaining stable for up to 10 years (*p* = 0.001). Significant visual gain after treatment was recorded in subfoveal, juxtafoveal and extrafoveal CNV locations, and we did not find any correlation between final visual outcome and any CNV location. Interestingly for this discussion, the aggressive treatment regimen of three monthly doses of IVB followed by PRN administered in our group would have been able to improve visual acuity even in subfoveal mCNV location, as per Ruiz Moreno et al. [29]. Multiple regression analysis proved that pretreatment BCVA was the most influential prognostic factor significantly correlated with visual final outcome (β = 0.88, *p* < 0.0001, R^2^: 0.75), in agreement with Traversi et al., Yang et al. and our previous results (β = 0.560, *p* = 0.001) [16,23,24].

These encouraging data were obtained with a limited number of injections (mean total number: 6.8 ± 6.31) over the 10-year study period. As reported in other studies on IVB treatment for mCNV, the majority of injections were concentrated in the first 12 months when CNV activity is higher (mean 5.1 ± 3.36 in the first year), whereas, after the first year, a very limited number of treatments per year were needed to control disease activity [15,16,24,26]. The number of treatments was not correlated with final visual acuity, and no predictive factors for the need for retreatment were identified. Similarly to Ohno-Matsui et al. [1] findings, 26.7% of participants developed mCNV in the fellow eye with mCNV presentation at approximately 7 years in three patients and 1 year in the remaining five patients. Yoshida et al. studied the natural course of mCNV and reported that after three years since disease onset, BCVA progressively deteriorated for at least another seven years because of myopic CRA development/enlargement, with a possible decrease of anti-VEGF efficacy [3]. This is in agreement with our results reporting CRA development or progression around the mCNV in as many as 80% of the eyes at 3 years and in all study eyes at 5 and 10 years after the onset of CNV. A gradual decline in BCVA after an initial period of IVB treatment was observed in several studies [11,12,13,18,19,20,21]. The present paper, with the greatest BCVA improvement recorded at 2 years of follow-up time and the majority of IVB performed in the first year of treatment, is in general agreement with the aforementioned. Nevertheless, our results demonstrated visual gain maintenance at the end of follow-up, with initial BCVA correlating independently with BCVA outcome. Moreover, retinal and choroidal thickness significantly reduced over time as the result of CRA progression but without correlation neither with VA outcome nor with the number of injections. These favourable results indicate that, despite CRA progression, IVB treatment can be considered as safe and effective therapy in the long-term in mCNV. Additionally, unlike Ahn et al., we did not observe any correlation between subfoveal/inferior choroidal thickness at baseline and incomplete mCNV regression at the end of follow-up in anti-VEGF treated eyes [30].

Older age has been associated with development of CRA or poor visual outcome [16,21,22,23]; however, the present study did not show significant contribution of age for the progression of disease. The relatively old average age of the current population study could explain the nonsignificant effect of age, in accordance with Oishi et al. and Kasahara et al. [18,26]. In the current work, CNV area and height significantly decreased at 10 years, with complete regression of mCNV lesion in 40% of subjects at the end of follow-up. The value of CNV size is debatable; according to Yang et al. and Lai et al., it is significantly associated with final visual outcome [24,27]. Moreover, Yang et al. observed an association between baseline CNV lesion size and CNV recurrences in eyes with mCNV treated with one single IVB followed by a pro re nata protocol [24]. On the contrary, in both the different sized series of Ruiz Moreno et al. and Oishi et al., pretreatment CNV lesion area was not significantly associated with final outcome [18,21]. In accordance, we did not observe any correlation between baseline CNV lesion size, need for retreatment and final visual acuity in eyes with mCNV. This could be attributable to the difference in IVB treatment schedule and in the type of carried out measurements.

The limitations of the present study lie in its retrospective design, the relatively limited number of participants and, finally, the involvement of a single ophthalmologic centre. Therefore, large clinical trials are firmly expected to validate our results.

## 4. Methods

### 4.1. Patients

The study consisted of a retrospective analysis of 30 consecutive treatment-naïve patients (30 eyes) with mCNV treated with IVB at the University Hospital ‘Sapienza’ of Rome and with a follow-up of at least 10 years. Each patient underwent complete clinical and instrumental ophthalmological examination at baseline and monthly as re-evaluations under PRN regimen, including: best-corrected visual acuity (BCVA) measurement, spectral domain optical coherence tomography (SD-OCT) and colour fundus photography. Fluorescein angiography (FA) was performed at baseline and whenever mCNV activity was suspected. Inclusion criteria were: 10 years of follow-up time; spherical equivalent > −6.0 dioptres or axial length > 26.5 mm (IOLMaster, version 4.07; Carl Zeiss Meditec, Dublin, CA, USA); subfoveal, juxtafoveal or extrafoveal CNV (if located under the geometric centre of the foveal avascular zone, <200 µm or >200 µm from the fovea, respectively); and evidence of disease activity on SD-OCT and FA. Exclusion criteria were: any previous treatment for mCNV, dome-shaped macula (DSM) and/or myopic traction maculopathy (MTM), retinal drusen or any evidence of dry/wet AMD, history of ocular surgery other than cataract extraction, any other ocular condition that could affect visual acuity, and any systemic condition resulting incompatible with the use of anti-VEGFs.

All patients’ medical charts and imaging were collected, and retrospective evaluation was conducted in detail. The following variables were recorded: age, axial length, BCVA, CNV type, location, max height and area, CNV lesion complete/incomplete regression, retinal and choroidal thickness maps at central 1 mm subfield (CSF), 3 mm and 6 mm areas of two peripheral rings from central point thickness, number of administered IVB injections and need for retreatment.

### 4.2. BCVA

BCVA was measured by using the standardized, 70-letter Early Treatment Diabetic Retinopathy Study chart (Chart ‘R’, Precision Vision, La Salle, IL, USA) at 4 m distance. Visual acuity was converted to decimal logarithm of the minimum angle of resolution (logMAR) for statistical analysis.

### 4.3. OCT Evaluations

SD-OCT images were acquired by using the Spectralis OCT (Spectralis Family Acquisition Module, V 6.0.11.0; Heidelberg Engineering, Heidelberg, Germany) through standardized protocol. Active Eye tracking technology and AutoRescan feature allowed for accuracy and reproducibility of scans and related measurements. Scan protocol consisted of a raster horizontal 20° × 15°, 19-line scan and a radial 20°, 6-line scan centred on the fovea to be collected for each eye in order to evaluate the presence of intraretinal or subretinal fluid, CNV type, location, max height and size and CNV lesion complete/incomplete regression.

The B-scan presenting the largest area of the CNV lesion was chosen for measuring the entire area and max height of the CNV lesion according to K. Abri Aghdam et al. and T. Ach et al., and the same scan was evaluated over time to assess morphological changes using the built-in eye tracker [30,31]. Macular and choroidal thickness values were automatically calculated by the built-in software. Macular thickness was delineated as the distance between the vitreoretinal interface and the outer border of the retinal pigment epithelium.

Mean retinal thickness values were displayed in sectors created by three concentric rings with diameters of 1 (central), 3 (inner) and 6 (outer) mm corresponding to the 9 subfields of the early treatment diabetic retinopathy study (ETDRS) map. The inner and outer rings were divided into 4 subfields. Retinal thickness within the inner circle (1 mm diameter) was defined as the central subfield (CSF) retinal thickness.

The same protocol was employed for the choroid, evaluating choroidal thickness in all the ETDRS subfields. Choroidal thickness measurements were conducted in a semiautomatic method, according to previous evidence [32,33]. The Bruch’s membrane and the choroid–scleral interface were considered landmarks. In order to further analyse retinal and choroidal thickness change over time, we compared the included patients with a group of 30 subjects affected by uncomplicated PM and followed-up with SD-OCT for at least 10 years. The control group was matched for age and axial length.

### 4.4. Colour Fundus Photography and FA

Colour fundus photography and FA angiograms were obtained with the Topcon fundus camera (TRC 50DX; Topcon, Tokyo, Japan). FA was performed according to standard procedure, involving the intravenous injection of 5 mL of 10% sodium fluorescein solution (Novartis Pharma AG, Bern, Switzerland). Leakage from the CNV was evaluated on FA in the late phase (6–8 min) compared with the early phase (10–20 s).

All mCNV were classified based on FA/OCT images at baseline evaluation [28]. CRA was considered as patchy and diffuse myopic atrophy adjacent to CNV based on colour fundus photography, FA and OCT images.

Instrumental evaluations were performed by certified optometrists and interpreted by 2 independent retinal specialists.

### 4.5. Treatments

All participants were treated with a loading dose of three monthly injections of IVB (1.25 mg/0.05 mL) at baseline and then evaluated and treated with additional IVB injections in case of persistence or recurrence of CNV according to PRN regimen. As previously described from our group, additional injections were indicated until disappearance of fluorescein leakage from the CNV and intraretinal/subretinal fluid on SD-OCT [16].

Patients who switched to intravitreal anti-VEGF agents other than bevacizumab were excluded from the present study. All intravitreal injections were performed by an experienced retinal specialist according to usual procedure, including topical anaesthesia and surface disinfection with 5% povidone–iodine solution. Intravitreal injection of 1.25 mg bevacizumab was performed through the pars plana, 3.5–4 mm from the limbus in the inferotemporal quadrant using a 30-gauge needle. Topical antibiotic was administered at the end of the procedure. The research followed the Tenets of the Declaration of Helsinki, and informed consent was obtained from all subjects of the study.

### 4.6. Statistical Analysis

Descriptive statistical analysis was performed in all collected data. Continuous variables were presented as mean ± standard deviation. Longitudinal data were analysed by the paired *t*-test, and intergroup differences were evaluated using the independent Student’s *t*-test with Levene’s correction. Categorical variables were expressed as frequencies and percentages, and Fisher’s exact test was used for comparison analysis.

The correlations between age, axial length, visual acuity, retinal and choroidal thickness, CNV max height and area and number of injections were calculated by using the Spearman Rho index or the Pearson analysis, as appropriate.

Multivariate regression analysis was conducted to evaluate the effect of each potential predictive factor on final BCVA and need for retreatment. Intraobserver and interobserver agreement was evaluated with the K Cohen Statistic.

The analysis was conducted using IBM^®^ SPSS^®^ Statistics for Windows^®^, version 24.0 (IBM Corp., Armonk, NY, USA), and values above the 95% confidence level (*p* < 0.05) were considered statistically significant.

## 5. Conclusions

This study aims to represent the first evaluation of all the main prognostic factors determining the efficacy and safety of IVB treatment for mCNV over a 10-year follow-up time. The long-term follow-up enabled us to study the therapeutic potential of IVB to influence the natural condition course progressively over time and to monitor the fellow eye of patients with pre-existing mCNV. We speculate that strict monitoring of the variables involved in the response of the lesion to specific IVB treatment in the long-term may have provided results of great impact in practice use.

## Figures and Tables

**Table 1:** Demographic and clinical characteristics of 30 patients (30 eyes) with mCNV treated with IVB.

Characteristics	Total (30 Patients/30 Eyes)
Age (years)	63.7 ± 7.6
Gender (F/M)	F20/M10
Caucasic	30/30 (100%)
Axial length (mm)	30.7 ± 1.2
Baseline BCVA (ETDRS in logMAR)	0.47 ± 0.42
Phakic/pseudophakic/aphakic	14(46.7%)/14(46.7%)/2(6.7%)
Subfoveal CNV	6/30 (20%)
Juxtafoveal CNV	14/30 (46.7%)
Extrafoveal CNV	10/30(33.3%)
Retreated CNV	14/30 (46.7%)
Complete lesion regression	12/30 (40%)
Total n. injections	6.8 ± 6.31
Fellow eye involvement	8/30 (26.7%)

Values are mean ± SD unless otherwise indicated. BCVA, best corrected visual acuity; CNV, choroidal neovascularisation; ETDRS, Early Treatment Diabetic Retinopathy Study.

**Table 2:** Change in number of IVB injections and incidence of CRA during the 10-year follow-up period.

Outcome	1 Year	2 Years	3 Years	5 Years	10 Years	Total Number
N° of injections (mean ± SD)	5.1 ± 3.36	1.0 ± 2.13	0.7 ± 0.82	0	0	6.8 ± 6.31
CRA around mCNV (N° eyes/%)	7/30 (23%)	20/30 (66%)	24/30 (80%)	30/30 (100%)	30/30 (100%)	

CRA, Chorioretinal atrophy; mCNV, myopic choroidal neovascularization.

**Table 3:** Visual outcomes after IVB treatment for mCNV.

Outcome	Baseline	2 Years	4 Years	6 Years	8 Years	10 Years
BCVA (ETDRS in logMAR)	0.47 ± 0.42	0.28 ± 0.33	0.19 ± 0.28	0.22 ± 0.26	0.18 ± 0.25	0.21 ± 0.29
BCVA change (ETDRS in logMAR)		0.19 ± 0.15	0.28 ± 0.14	0.25 ± 0.16	0.29 ± 0.17	0.26 ± 0.22
*p* value		<0.0001 *				0.001 *

Values are mean ± SD unless otherwise indicated. * Student *t*-test for paired data. BCVA, best corrected visual acuity; ETDRS, Early Treatment Diabetic Retinopathy Study.

**Table 4:** Multivariate regression analysis to evaluate the contribution of each pretreatment factor on final BCVA at 10 years.

	BVCA at 10 Years		
Factor	B	β	*p* Value
BCVA T0 (No of ETDRS letters in logMAR)	0.602	0.876	<0.0001
Age (years)	0.044	0.796	0.851
CSF CT T0	−0.125	0.481	0.754
CSF RT T0	0.138	0.368	0.994
CNV A T0	0.005	0.978	0.929
CNV LOC	−0.032	0.847	0.892
Adjusted R^2^	0.747		

β, standardised regression coefficient; adjusted R^2^, coefficient of multiple determination; B, nonstandardised regression coefficient. BCVA, best corrected visual acuity; ETDRS, Early Treatment Diabetic Retinopathy Study; CSF, central subfield; CNV, choroidal neovascularization; CSF CT T0, CSF choroidal thickness at T0; CSF RT T0, CSF retinal thickness at T0; CNV A T0, CNV area at T0; CNV LOC, CNV location.

**Table 5:** Anatomical outcomes after IVB treatment for mCNV.

Outcome	Baseline	5 Years	10 Years	*p* Value 5 y	*p* Value 10 y
CNV A	0.12 ± 0.11	0.054 ± 0.06	0.044 ± 0.06	*p* = 0.017 *	*p* = 0.003 *
CNV H	165.53 ± 108.27	72.4 ± 67.44	59.53 ± 61.29	*p* < 0.0001 *	*p* < 0.0001 *
CSF CT	76.53 ± 39.35	58.53 ± 29.60	55.2 ± 31.81	*p* = 0.029 *	*p* = 0.036 *
CT3mm	70.43 ± 32.29	58.91 ± 29.43	60.18 ± 30.13	*p* = 0.004 *	*p* = 0.084 *
CT6mm	70.65 ± 27.14	61.56 ± 28.47	58.43 ± 26.74	*p* = 0.03 *	*p* = 0.002 *
CSF RT	277.2 ± 41.40	250.2 ± 38.09	218.06 ± 74.46	*p* = 0.01 *	*p* = 0.007 *
RT3mm	292.91 ± 33.22	271.12 ± 32.31	242.01 ± 61.69	*p* = 0.014 *	*p* = 0.003 *
RT6mm	250.5 ± 31.22	237.31 ± 30.12	203.57 ± 57.1	*p* = 0.145 *	*p* = 0.011 *

Values are mean ± SD unless otherwise indicated * Student *t*-test for paired data. CNV A, CNV area; CNV H, CNV height; CSF CT, central subfield choroidal thickness; CT3mm, choroidal thickness at 3mm from the fovea; CT6mm, choroidal thickness at 6 mm from the fovea; CSF RT, central subfield retinal thickness; RT3mm, retinal thickness at 3 mm from the fovea; RT6mm, retinal thickness at 6 mm from the fovea.

**Table 6:** Comparative analysis between patients and controls.

Outcome	Baseline CNV	Baseline no CNV	*p* Value Baseline	5 Years CNV	5 Years no CNV	*p* Value 5 Years	10 Years CNV	10 Years no CNV	*p* Value 10 Years
CSF CT	76.53 ± 39.35	65.25 ± 23.66	0.469 *	58.53 ± 29.60	46.63 ± 27.34	0.357 *	55.2 ± 31.81	32.62 ± 22.24	0.090 *
CSF CT change from baseline							−21.33 ± 35.56	−32.62 ± 20.74	0.421 **
CT3mm	70.43 ± 32.29	67.31 ± 35.05	0.832 *	58.91 ± 29.43	50.81 ± 28.63	0.533 *	60.18 ± 30.13	38.94 ± 22.67	0.096 *
CT3mm change from baseline							−10.25 ± 21.36	−28.37 ± 20.38	0.062 **
CT6mm	70.65 ± 27.14	69.31 ± 29.76	0.914 *	61.56 ± 28.47	50.12 ± 16.69	0.311 *	58.40 ± 26.74	36.57 ± 14.29	0.045 *
CT6mm change from baseline							−12.25 ± 12.56	−32.74 ± 28.57	0.088 **
CSF RT	277.2 ± 41.40	274.13 ± 39.70	0.865 *	250.2 ± 38.09	254.13 ± 50.58	0.836 *	218.06 ± 74.46	232.5 ± 39.62	0.617 *
CSF RT change from baseline							−59.13 ± 72.36	−41.62 ± 42.62	0.539 **
RT3mm	299.58 ± 36.97	291.53 ± 26.07	0.591 *	271.12 ± 32.31	263.1 ± 44.11	0.621 *	242.01 ± 61.69	248.03 ± 36.25	0.804 *
RT3mm change from baseline							−57.56 ± 50.14	−43.5 ± 27.68	0.473 **
RT6mm	250.5 ± 31.22	242.69 ± 35.21	0.590 *	237.32 ± 30.12	208.9 ± 53.97	0.117 *	203.57 ± 57.1	188.91 ± 70.07	0.593 *
RT6mm change from baseline							−46.93 ± 62.27	−53.78 ± 63.50	0.805 **

Values are mean ± SD unless otherwise indicated. * Unpaired *t*-test comparing patients and control thickness measurements. ** Unpaired *t*-test comparing patients and controls thickness change from baseline to 10 years. CSF CT, central subfield choroidal thickness; CT3mm, choroidal thickness at 3 mm from the fovea; CT6mm, choroidal thickness at 6 mm from the fovea; CSF RT, central subfield retinal thickness; RT3mm, retinal thickness at 3 mm from the fovea; RT6mm, retinal thickness at 6 mm from the fovea.

## Data Availability

Data is contained within the article.
